# Preparation and Characterization of Electrospun Poly(lactic acid)/Poly(ethylene glycol)–*b*–poly(propylene glycol)–*b*–poly(ethylene glycol)/Silicon Dioxide Nanofibrous Adsorbents for Selective Copper (II) Ions Removal from Wastewater

**DOI:** 10.3390/membranes13010054

**Published:** 2023-01-01

**Authors:** Muhammad Omer Aijaz, Seong Baek Yang, Mohammad Rezaul Karim, Ibrahim Abdullah Alnaser, Abdulelah Dhaifallah Alahmari, Fahad S. Almubaddel, Abdulaziz K. Assaifan

**Affiliations:** 1 Department of Mechanical Engineering, College of Engineering, King Saud University, Riyadh 11421, Saudi Arabia; 2 Department of Chemical Engineering, College of Engineering, King Saud University, Riyadh 11421, Saudi Arabia; 3 Department of Biomedical Technology, College of Applied Medical Sciences, King Saud University, Riyadh 11421, Saudi Arabia

**Keywords:** poly(lactic acid), poly(ethylene glycol)-*b*-poly(propylene glycol)-*b*-poly(ethylene glycol), silicon dioxide, electrospinning, nanofibrous adsorbents, heavy metal adsorption

## Abstract

The problem of industrial wastewater containing heavy metals is always a big concern, especially Cu^2+^, which interprets the soil activity in farmland and leaves a negative impact on the environment by damaging the health of animals. Various methods have been proposed as countermeasures against heavy-metal contaminations, and, as a part of this, an electrospun nanofibrous adsorption method for wastewater treatment is presented as an alternative. Poly(lactic acid) (PLA) is a biopolymer with an intrinsic hydrophobic property that has been considered one of the sustainable nanofibrous adsorbents for carrying adsorbate. Due to the hydrophobic nature of PLA, it is difficult to adsorb Cu^2+^ contained in wastewater. In this study, the hydrophilic PLA/poly(ethylene glycol)-poly(propylene glycol)-poly(ethylene glycol) (PEG-PPG-PEG) nanofibrous adsorbents with different silicon dioxide (SiO_2_) concentrations were successfully prepared by electrospinning. A hydrophilic group of PEG-PPG-PEG was imparted in PLA by the blending method. The prepared PLA/PEG-PPG-PEG/SiO_2_ nanofibrous adsorbents were analyzed with their morphological, contact angle analysis, and chemical structure. The Cu^2+^ adsorption capacities of the different PLA/PEG-PPG-PEG/SiO_2_ nanofibrous adsorbents were also investigated. The adsorption results indicated that the Cu^2+^ removal capacity of PLA/PEG-PPG-PEG/SiO_2_ nanofibrous adsorbents was higher than that of pure ones. Additionally, as an affinity nanofibrous adsorbent, its adsorption capacity was maintained after multiple recycling processes (desorption and re-adsorption). It is expected to be a promising nanofibrous adsorbents that will adsorb Cu^2+^ for wastewater treatment.

## 1. Introduction

Global water demand is increasing at a rate of more than 3% every year, and the water shortage is causing political and economic problems in many parts of the countries (e.g., Malta, Algeria, Jordan, Maldives, Saudi Arabia, Libyan Arab Jamahiriya, United Arab Emirates, Bahrain, Kuwait, Yemen, Qatar, Morocco) [1,2]. If problems arise at this rate, it can cause major problems with the supply of fresh water to households. In recycling fresh water, it is important to purify wastewater. Among them, the removal of heavy metals is significant for human health. The removal of toxic metal ions from industrial wastewater has received a lot of attention in recent years to preserve the health of living organisms. The scientific community and industry are also making great efforts to solve this problem. Manufacturing industries such as paper, electricity, electronics, textiles, plastics, and dyes consume significant amounts of water and use chemicals containing heavy metals, which are palladium, platinum, copper, iron, chromium, arsenic etc. As a result, these industries create a considerable amount of contaminated wastewater; among heavy metals, copper (Cu) is one of the most used elements in the production industries such as electrical, antifouling, and paint industries. It has many toxic effects on human health including cancer, liver damage, Wilson disease, and insomnia [3,4].

Various techniques, such as reverse osmosis [5], chemical and electrochemical treatments [6,7], and solvent extraction and adsorption [8,9] have been used in the past to remove heavy metals from water. Each method has its own advantages and disadvantages; amongst the methods, adsorption as a highly efficient and facile method is an ideal option for the removal of toxic heavy metal ions from water due to its ease of operation, use of tailored adsorbents, and controlled design [10]. Electrospun nanofibrous adsorbents attracted great interest in the field of water purification due to their ultra-high specific surface area, nano-order thickness, and ultra-highly oriented molecule. By electrostatically drawing polymer solution jets, the electrospinning apparatus produces electrospun nanofibrous adsorbents. Droplets of polymer solution are stretched under high voltage to form nano or microfibers, which are then deposited on the collector [9]. The extracellular matrix in electrospun nanofibrous adsorbents gives them their high porosity, small pore size, and suitable mechanical properties [11]. Compared to conventional technology, the main advantage of electrospun nanofibers is the ability to remove undesirable particles and elements from water at a low cost (mainly saving on electricity) [12].

**Figure 1:**
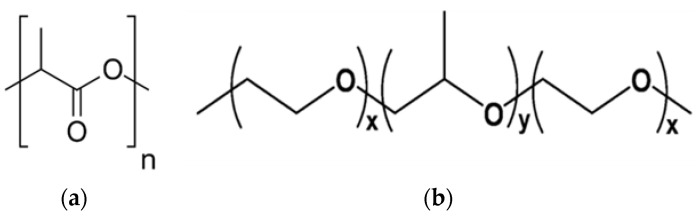
Chemical structures of PLA (**a**) and PEG–*b*–PPG–*b*–PEG (**b**) block copolymer.

Poly(lactic acid) (PLA) is a hydrophobic, biocompatible, and biodegradable polymer that has great protection from water, and the film it produces is likewise hydrophobic due to the structural configuration as shown in Figure 1a [13,14]. The adsorbent surfaces applied to remove heavy metal ions have been classified into hydrophobic surfaces at a contact angle higher than 90° and hydrophilic surfaces at a lower contact angle of 90° [15]. Super hydrophilic exhibit once the contact angle of the adsorbent surfaces is lower than 10°. On a super hydrophilic surface, water tends to form a thin film rather than droplets [16], which helps the adsorbent to capture the maximum amount of pollutants on its surface. PLA’s surface is highly hydrophobic, although many surface and bulk changes have been found to improve hydrophilicity; thus, coating, grafting, and blending PLA with more hydrophilic polymers is used to modify the surfaces of PLA [17]. An important factor needs to be taken into account when using biopolymer as an adsorber. Materials must be carefully chosen because surface and bulk polymer properties can both affect the adsorption capacity strength of the heavy metal ions [18,19]. For instance, the polymer matrix needs to be sufficiently hydrophilic to permit wetting and subsequent penetration of the material by aqueous media. Additionally, the polymer matrix cannot be excessively water soluble such that it dissolves or swells when aqueous media are present.

The biocompatibility and functionality of materials both significantly improve when surface wetting is improved [20,21]. Nanoparticles (NPs) in electrospun structures are frequently used in adsorption applications. NPs materials have a larger surface area than bulk materials, which makes it possible to capture heavy metals in aqueous media more successfully. Therefore, it is important to improve the surface hydrophilicity of the nanoscale materials. Generally, improvements in surface hydrophilicity can be monitored by measuring wettability by contact angle [11,20].

Several works published adsorption of toxic metal ions was improved by incorporating NPs such as iron oxide, zinc oxide (ZnO), titanium dioxide, silicon dioxide (SiO_2_), etc., into the electrospun nanofibrous adsorbents [22,23,24,25]. Makaremi et al. [26] reported the improvement of the chromium ion removal efficiency by incorporating ZnO into polyacrylonitrile (PAN) nanofibrous adsorbents. Another study incorporated the iron particles into the polyetherimide-based nanofibers and successfully applied for the removal of nickel ions. The SiO_2_ can be configured on electrospun nanofibrous adsorbents, since SiO_2_ possess superior biocompatibility, hydrophobicity, material matrix stability, and wide range of functionality. Previously, in another study, polyvinylpyrrolidone electrospun nanofibers were coupled with SiO_2_ for the adsorption of several metal ions removal from aqueous solution [27].

The main novelty of this study is the investigation of the prepared PLA/PEG-PPG-PEG/SiO_2_ nanofibrous adsorbents for their capacity to adsorb Cu^2+^ from an aqueous system. The main goals were to convert the hydrophobicity of PLA into hydrophilicity by blending of PEG-PPG-PEG, which contains both hydrophilic (PEG) and hydrophobic (PPG) groups, as shown in Figure 1b, followed by incorporating of SiO_2_ to enhance adsorption capacity through increasing surface area. The PLA/PEG-PPG-PEG/SiO_2_ nanofibrous adsorbents were then analyzed to assess their characteristics and potential as an effective adsorbent to remove Cu^2+^ from aqueous solutions. By studying kinetics (pseudo-first-order, pseudo-second-order, Elovich, power function, and intraparticle diffusion) and isotherm models (Langmuir, Freundlich and Temkin isotherm models) for adsorption, the Cu^2+^ adsorption capacities of the different PLA/PEG-PPG-PEG/SiO_2_ nanofibrous adsorbents were also investigated.

## 2. Materials and Methods

### 2.1. Materials

PLA(LX175^®^) purchased from Filabot Co., Ltd. (Barre, VT, USA) and PEG-PPG-PEG (Pluronic^®^ F-108) purchased from Sigma Aldrich, (St. Louis, MO, USA) were used as a polymer, which is a major component of the flat-sheet nanofibrous adsorbents; additionally, SiO_2_ purchased from Sigma Aldrich, which is a nanomaterial to be incorporated, was used. To prepare a solution for electrospinning, the following materials, as a solvent, were used: dichloromethane (DCM) and dimethylformamide (DMF) purchased from Sigma Aldrich without purification. To check the adsorption of heavy metal’s remove ability, used copper sulfate and hydrochloric acid (HCl) were purchased from Sigma Aldrich. Additionally, all water used in this study was deionized water.

### 2.2. Preparation of Electrospun PLA/PEG-PPG-PEG/SiO_2_ Nanofibrous Adsorbents

The PLA and PEG-PPG-PEG solutions were initially prepared separately. First, 12.5% (*w*/*v*) of PLA powder was dissolved in DCM at 50 °C for 1 h, and then 9% (*w*/*v*) of PEG-PPG-PEG powder was dissolved in DMF at 50 °C for 30 min [17]. After preparing each solution, both the solutions were blended in the 4:1 ratio for PLA and PEG-PPG-PEG solutions, respectively, and kept for 12 h of stirring to prepare an absolutely homogenized solution. To prepare PLA/PEG-PPG-PEG/SiO_2_ dope solutions, different SiO_2_ nanoparticles (1, 2, 3, 4, and 8 %*w*/*w*) were added in the prepared PLA/PEG-PPG-PEG blend solution. Prior to the electrospinning process, the PLA/PEG-PPG-PEG/SiO_2_ blend solution was magnetically stirred for 24 h to ensure homogeneity and dispersion. An electrospinning syringe was filled with the doped PLA/PEG-PPG-PEG/SiO_2_ blended solution. Electrospinning is carried out at a 25 °C of temperature and 10% of humidity. Lastly, the prepared nanofibrous adsorbents were carefully placed in an oven at 50 °C for 4 h before further characterization. Then, it was labelled and the collected nanofibrous adsorbents were kept according to Table 1.

### 2.3. Characterization of Prepared PLA/PEG-PPG-PEG/SiO_2_ Nanofibrous Adsorbents

The morphologies of the prepared nanofibrous adsorbents listed in Table 1 were examined qualitatively by field emission scanning electron microscopy (FE-SEM, JSM-7600, JEOL, Tokyo, Japan), and the energy dispersion X-ray (EDX) of the nanofibrous adsorbents was also taken using the EDX available with the FE-SEM analysis. To analyze the samples by FE-SEM and EDX, the platinum coating was performed under a vacuum for the 60 s. Surface morphological images were taken at ×5000 magnifications. The contact angle goniometer (OCA15EC, Data physics) was used to study the wettability behavior of prepared nanofibrous adsorbents. The 5 μL droplets of deionized water were positioned on the nanofibrous adsorbents to measure the droplet angle between the liquid and nanofibrous adsorbents surface. An average of at least ten water contact angle (WCA) measurements are observed at different places for each sample. When a droplet was dropped for 50 s from the beginning, the contact angle was measured and then plotted. To check the chemical vibration of the prepared nanofibrous adsorbents depending on various SiO_2_ contents, it is measured by Fourier transform infrared (FT-IR, Bruker, Billerica, MA, USA) spectroscopy in the wavelength of 550–4500 cm^−1^ to identify the chemical structure of the nanofibrous adsorbents. To confirm that PLA/PEG-PPG-PEG/SiO_2_ was synthesized and spun, X-ray diffraction (XRD) analysis (D/Max–2500, Rigaku, Tokyo, Japan) was performed.

### 2.4. Removal of Heavy Metals by Adsorption Process

The stock solution of Cu^2+^ for the adsorption study was prepared by adding 1000 mg of CuSO_4_·5H_2_O into 1000 mL of deionized water. For the adsorption study, certain amounts of nanofibrous adsorbents were immersed in Cu^2+^ containing solution and shaken at 25 °C. Finally, Cu^2+^ solution concentration after the adsorption studies were measured by atomic absorption spectroscopy (AAS, AA-7000, Tokyo, Japan). A few actions were taken before the samples were analyzed using AAS. Aspirating blank solution and adjusting zero were done first, and then at least three concentrations of prepared Cu^2+^ standard solutions were selected to be examined. Each standard solution should be aspirated into the flame to calibrate the AAS system. Unknown samples were aspirated after the machine generated the standard curve, the reading of the ready sample solution was taken directly from the instrument, and the adsorption capacity of metal ions was calculated using the below equation.

$$Q=\frac{C_{0}-C_{E}}{M\times V}$$

where Q is the amount of metal ions (Cu^2+^) adsorb in milligram (mg/g), $C_{0}$ and $C_{E}$ was the initial and final concentration of metal ions, respectively, in part per million (ppm), V was the metal ion solution volume in liter (L), and M was the mass of adsorbent used in gram (g). Reported adsorption data were calculated using the average of three triplets.

To study the influence of pH (4–6), time (15–480 min), and concentration (10–400 ppm) on the adsorption of Cu^2+^, approximately 18 mg of nanofibrous adsorbents were cut into small pieces and placed in a vial containing 15 mL of Cu^2+^ solution while being shaken at 300 rpm. The pH values were adjusted using 0.5 M of HCl. Due to the metal hydroxide precipitations, the effects of pH at higher values were not observed [28,29,30]. After the tests were completed, the samples were removed from the vials with a tweezer and dried in an electric oven for reusability testing.

### 2.5. Kinetics and Isotherm Models for Adsorption Study

Different kinetic models, including pseudo-first-order (PFO), pseudo-second-order (PSO), Elovich, power function, and intraparticle diffusion, were used to better explore the adsorption mechanisms onto nanofibrous adsorbents during the process of adsorption. The standard error of estimate (SEE) and coefficient of determination (R^2^) were computed to assess the degree of agreement between the experimental and model-predicted adsorption data. Table 2 and Table 3 show the linear expressions of the aforementioned estimations and kinetics, respectively.

### 2.6. Recyclability Study of the Prepared Nanofibrous Adsorbents

For the reusability test, cleaning of Cu^2+^ ions from the nanofibrous adsorbent’s surface of the maximum adsorption capacity adsorbent (#3S) was carried out by washing in 0.1 M of HCl aqueous solution for 1 h. The washed sample was then separated from the acid solution, washed several times with distilled water, dried, and reused for further adsorption processes [38,39]. This process was repeated four times by using the same adsorbent in a batch experiment.

## 3. Results and Discussion

### 3.1. Preparation of the Electrospun PLA/PEG-PPG-PEG/SiO_2_ Nanofibrous Adsorbents

#### 3.1.1. Morphological Analysis of PLA/PEG-PPG-PEG/SiO_2_ Nanofibrous Adsorbents

**Figure 2:**
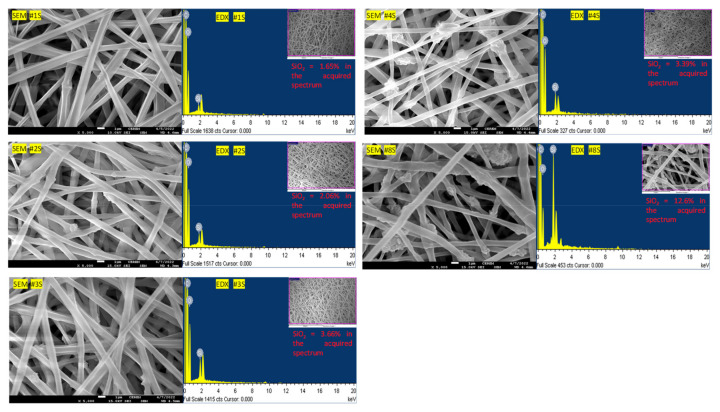
FE-SEM images and EDX results of the electrospun PLA/PEG-PPG-PEG/SiO_2_ nanofibrous adsorbents containing the various concentrations of SiO_2_.

The FE-SEM and EDX of electrospun PLA/PEG-PPG-PEG/SiO_2_ nanofibrous adsorbents with various SiO_2_ concentrations of 1, 2, 3, 4, and 8 %*w*/*w* corresponding to #1S, #2S, #3S, #4S and #8S, respectively, is presented in Figure 2. Incorporating the concentration of SiO_2_ was 1–4 %*w*/*w* (#1S-#4S); a similar diameter of nanofibers was generally prepared. The electrospun PLA/PEG-PPG-PEG/SiO_2_ nanofibers were prepared in the shape of nodes in bamboo. The concentration of SiO_2_ was 8 %*w*/*w* (#8S); some of the electrospun PLA/PEG-PPG-PEG/SiO_2_ nanofibers were coarse fibers, and it was prepared in non-uniform form. This trend is like any electrospun nanocomposites nanoweb incorporating nanoparticles. The content of SiO_2_ could be directly confirmed through the EDX analysis result. In general, electrospun nanofibers were observed as much as they were incorporated. As a result, it was confirmed that the preparation of PLA/PEG-PPG-PEG/SiO_2_ nanofibrous adsorbents was successfully performed.

#### 3.1.2. Contact Angle of PLA/PEG-PPG-PEG/SiO_2_ Nanofibrous Adsorbents

**Figure 3:**
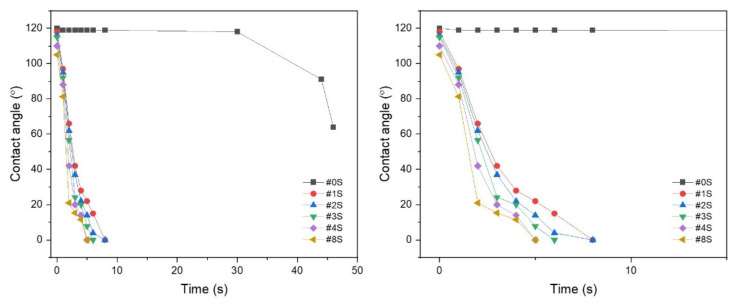
Contact angle the electrospun PLA/PEG-PPG-PEG/SiO_2_ nanofibrous adsorbents containing the various concentrations of SiO_2_.

Figure 3 shows the initial measurement value of the contact angle according to the SiO_2_ content and the tendency of the contact angle to decrease as time continues. In the case of the PLA/PEG-PPG-PEG blended nanofibers, it was confirmed that the contact angle was initially 120°and decreased to 60 degrees over time. In the case of the PLA/PEG-PPG-PEG/SiO_2_ blended nanofibrous adsorbents containing SiO_2_, it was confirmed that the contact angle became 0° within 10 s. This is because SiO_2_ exhibits hydrophilicity and attracts water molecules better. In particular, it was confirmed that the time for the contact angle to decrease to 0° decreased as the content of SiO_2_ increased. This means that the hydrophilic property increases as the content of SiO_2_ in the nanofibers increases. It is thought that as the hydrophilicity increases, the effective contact surface for heavy metal adsorption can be increased.

#### 3.1.3. Chemical Structure Analysis of PLA/PEG-PPG-PEG/SiO_2_ Nanofibrous Adsorbents

**Figure 4:**
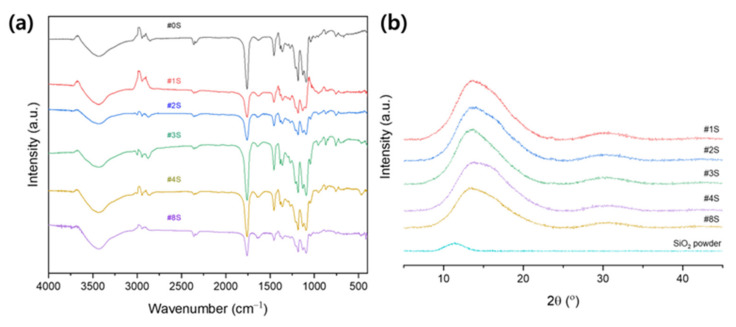
(**a**) FT-IR spectra and (**b**) XRD results of the SiO_2_ powder and the electrospun PLA/PEG-PPG-PEG/SiO_2_ nanofibrous adsorbents containing various concentrations of SiO_2_.

Figure 4a shows FT-IR spectra of PLA/PEG-PPG-PEG nanofibrous adsorbents and PLA/PEG-PPG-PEG/SiO_2_ nanofibrous adsorbents containing various SiO_2_ concentration (1, 2, 3, 4, and 8 %*w*/*w*). PLA exhibits characteristic stretching frequencies for C=O, –CH_3_ asymmetric, –CH_3_ symmetric, and C–O at 1746, 2995, 2946, and 1080 cm^−1^, respectively. It has been determined that the bending frequencies for –CH_3_ asymmetric and –CH_3_ symmetric are 1452 and 1361 cm^−1^, respectively [40,41]. In the spectrum of the PLA/PEG-PPG-PEG blended nanofibers, the absorption band at 3487 cm^−1^ was attributed to the hydroxyl groups (–OH) of PEG-PPG-PEG and PLA chains [40,42,43]. A weak absorption peak appears at 2860 and 2970 cm^−1^ is attributed to the stretching vibration peak of CH_2_ and C–H in PEG-PPG-PEG copolymer [44,45]. In the spectrum of PLA/PEG-PPG-PEG/SiO_2_ nanofibers, all the peaks identified in PLA/PEG-PPG-PEG were found; however, the shape was slightly different depending on the content of SiO_2._ Moreover, the presence of Si–O–Si stretching vibration bonding at 1093, 798, and 459 cm^−1^ were revealed [46]. It is also found that some peaks have shift or change with the increase of SiO_2_. This indicates that neither a strong chemical interaction nor the formation of a new bond took place within the blend and nanoparticles. Through each peak, these results show that it is successfully obtained PLA/PEG-PPG-PEG/SiO_2_ nanofibrous adsorbents. Because the electrospun PLA/PEG-PPG-PEG/SiO_2_ nanofibrous adsorbents have –OH group of PEG-PPG-PEG, it is hydrophilic. Due to SiO_2_ also being hydrophilic material, then the prepared PLA/PEG-PPG-PEG/SiO_2_ nanocomposite nanofibrous adsorbents also seems hydrophilic.

Figure 4b shows the XRD patterns of pure SiO_2_ powder and PLA/PEG-PPG-PEG/SiO_2_ nanofibers containing varying concentrations of SiO_2_ (1, 2, 3, 4, and 8 %*w*/*w*). PLA’s amorphous microstructure is confirmed by the broad amorphous peaks at around 16.8° in PLA/ PEG-PPG-PEG [47]. The characteristics peaks of PEG (2θ = 19.2° and 23.2°) [48] and a typical amorphous halo at 21° [49] for PPG did not appear in PLA/PEG-PPG-PEG composite nanofibrous adsorbents due to the low ratio of PEG and PPG with respect to matrix polymer PLA as well as good dispersion into the parent PLA composite nanofiber. This suggested that composite crystallinity first increased and then decreased with increased SiO_2_ mass ratio. This was due to the agglomeration of SiO_2_, resulting in the reduction of PLA nucleation effects and the number of effective crystal nuclei.

### 3.2. Evaluation of Nanofibrous Adsorbents in Adsorption Applications

#### 3.2.1. Effect of pH on Adsorption Capacity

**Figure 5:**
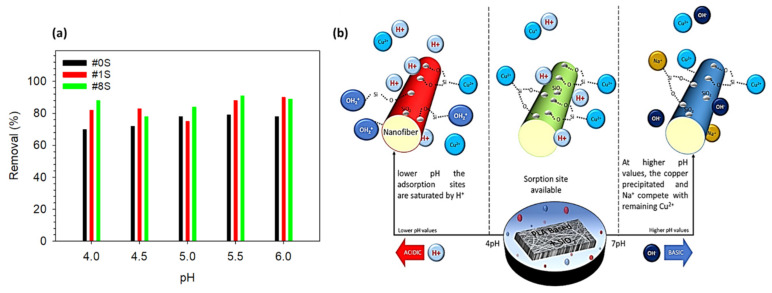
(**a**) The impact of pH on the adsorption of Cu^2+^ onto PLA-based nanofibrous adsorbents. (**b**) Schematic illustration of the adsorption of a heavy metal (in this case, copper) onto a prepared nanofibrous adsorbents. At lower pH values, H^+^ saturates the adsorption sites and copper ion adsorption is low. The availability of sorption sites and the adsorption of copper ions both increase as the pH rises. Copper precipitated and Na^+^ competed with any remaining Cu^2+^ at high pH values.

The effect of pH on the adsorption efficiency of PLA-based nanofibrous adsorbents is discussed in this section. The pH level of the metal ions solution is crucial in regulating the amount of ions that are adsorbed onto the adsorbent material [50]. For checking the effect of pH, 10 ppm Cu^2+^ solutions were added with suitable amounts of HCl to adjust the pH from 4 to 6. The 18 mg of #0S, #1S, and #8S nanofibrous adsorbents were poured in the pH-adjusted prepared solutions for 15 min at room temperature. All nanofibrous adsorbents showed an increase in the percentage of Cu^2+^ removal as the pH value rose from 4 to 6. The maximum removal percentage for the #8S sample at pH 5.5 is shown in Figure 5a. Due to the increased surface area created by the addition of SiO_2_ nanoparticles, the removal percentage for the #8S was higher when compared to less or one without SiO_2_.

In numerous studies, the adsorption of Cu^2+^ on adsorbates is highest in the pH range of 4–6, and it is reported that the pH above 7 was avoided because the alkaline solution’s insoluble metal hydroxide precipitates blocked the reaction sites’ active sites [30]. Low pH values also are avoided, as at lower pH values, the adsorption sites are saturated due to protonation by H^+^ and the adsorption of copper ions is low. When the pH increases from 4 to 6, the amount of H^+^ ions decrease, which reduces protonation around SiO_2_ and increases Cu^2+^ adsorption because there are more sorption sites available for Cu^2+^ [27]. The effect of pH on the adsorption of Cu^2+^ onto PLA based nanofibrous adsorbents is presented schematically in Figure 5b [51,52,53,54].

#### 3.2.2. Effect of Time Interval on Adsorption Capacity

**Figure 6:**
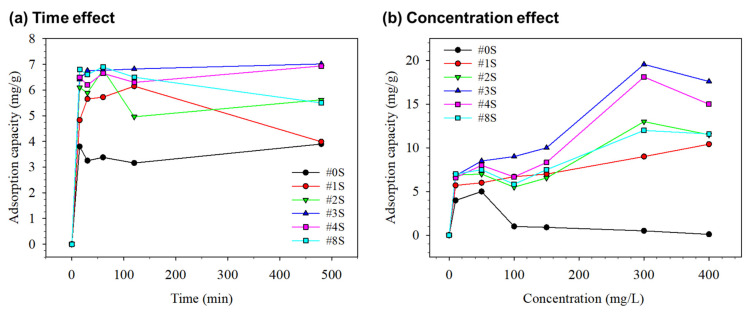
(**a**) Time interval and (**b**) concentration’s effect of adsorption of Cu^2+^ on various samples.

Figure 6a shows the effect of adsorption time on the adsorption capacity of PLA/PEG-PPG-PEG/SiO_2_ nanofibrous adsorbents containing various SiO_2_ contents such as #0S, #1S, #2S, #3S, #4S, and #8S. The study of effect of time on adsorption capacity was performed using 15 mL of initial Cu^2+^ at a concentration of 10 mg/L with 18 mg of all mentioned nanofibrous adsorbents at a pH 5.5 for 1–480 min. As the time passed until 15 min, the adsorption capacity sharply rose. After 15 min, the rate of adsorption growth started to slow down, and, after 60 min, it reached an equilibrium. The presence of free sites, increased surface area, and porosities of the nanofibrous adsorbents may have contributed to the sharp increase in adsorption rate that occurred after 15 min. After 15 min, the rate of adsorption capacity growth began to slow down as contact time increased, eventually reaching an equilibrium. This phenomenon was brought on by the concentration of metal ions and the limited availability of adsorptive sites [55]. All adsorbents showed a similar effect of time on adsorption, but the adsorbent containing SiO_2_ had a higher adsorption capacity. This could be attributed to the presence of SiO_2_ particles, which increased the specific surface area and adsorption-free active sites.

#### 3.2.3. Effect of Concentration on Adsorption Capacity

Figure 6b shows the effect of the initial concentration of the adsorption of Cu^2+^ onto PLA/PEG-PPG-PEG/SiO_2_ nanofibrous adsorbents (such as #0S, #1S, #2S, #3S, #4S, and #8S). Through concentration experiments with 15 mL of initial Cu^2+^ concentration (10–400 mg/L) and 18 mg of nanofibrous adsorbents, the maximum adsorption of metal ions at pH 5.5 and 60 min was observed. The adsorption capacity gradually improved as the initial Cu^2+^ concentration rose. The prepared #3S and #4S nanofibrous adsorbents exhibited a higher Cu^2+^ adsorption capacity than nanofibrous adsorbents with less SiO_2_ quantity. The SiO_2_ nanoparticles inside the adsorbent provided more Cu^2+^ adsorption sites, which contributed to the increased adsorption of #3S and #4S [27]. As the initial concentration changed from 10 to 150 mg/L, the adsorption capacity rose dramatically. When the initial concentration reached 350 mg/L, the adsorption tendency slowed after 150 mg/L and reached an equilibrium value of roughly 19.56 mg/g (#3S) and roughly 18.12 mg/g (#4S). This concentration study also showed that #3S performed more effectively as an adsorbent for Cu^2+^, with a dominant effect, than the other adsorbent. The increased rate of Cu^2+^ adsorption on the #3S and #4S adsorbents may be caused by Si-O functional groups of silicon dioxide [55]. A slight fall in adsorption capacity was observed with higher copper ions concentrations (i.e., >400 mg/L), which could be attributed to agglomeration and steric hindrance effects [56].

### 3.3. Kinetics and Isotherm Models for Adsorption Study

**Figure 7:**
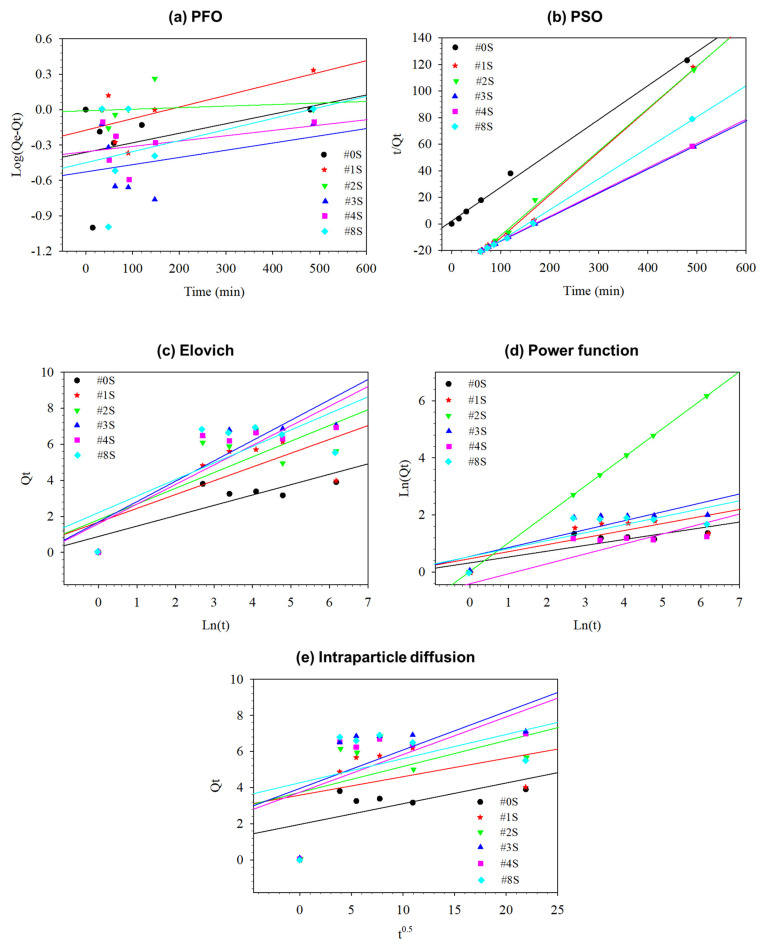
Plot lines showing the kinetic models for (**a**) PFO, (**b**) PSO, (**c**) Elovich, (**d**) Power function, and (**e**) Intraparticle diffusion.

The plotted kinetic models are shown in Figure 7, and Table 4 lists the calculated parameters as well as the R^2^ and SEE values for the Elovich, power function, intraparticle diffusion, and PFO and PSO models. All of the prepared composite nanofibrous adsorbents displayed pseudo-second-order R^2^ values that were closer to one, demonstrating that PSO was preferable to PFO for all of the nanofibrous adsorbents because PSO indicated that the adsorption process was chemisorption and involved an exchange or share of electrons between the adsorbent and adsorbate [31]. A further confirmation of the chemisorption nature of nanofibrous adsorbents was provided by the high value of Elovich (α) parameters [32,33]. The boundary layer effect was also a part of the adsorption process, as shown by the high value of the intra-particle diffusion parameter c. The power function model’s estimated rate coefficient (K_f_) value was higher, suggesting increased adsorption amount of Cu^2+^ with time.

**Figure 8:**
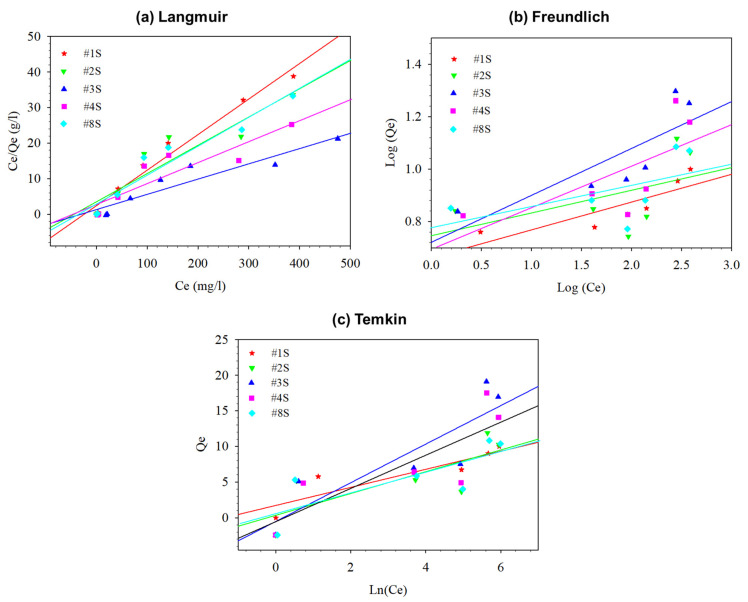
The plotted isotherm models of (**a**) Langmuir, (**b**) Freundlich, and (**c**) Temkin.

The Cu^2+^ adsorption equilibrium data of prepared PLA/PEG-PPG-PEG/SiO_2_ nanofibrous adsorbents retrieved from the concentration study and analyzed using Langmuir, Freundlich, and Temkin isotherm models. For the Langmuir, Freundlich, and Temkin models, the equations of isotherm models were listed in Table 2. Figure 8 and Table 5 both display the fitted lines of the employed models as well as a summary of the calculated parameters and error functions.

When comparing the Langmuir and Freundlich models, the calculated parameters indicated that the Langmuir model provided a better fit. Particularly, sample #3S demonstrated a high R^2^ value (roughly 0.9) with concordant SEE values. The Temkin model values suggested that the amount of adsorption had a negligible effect on heat compared to the Langmuir model. The Langmuir model suggested a monolayer adsorption of Cu^2+^ onto the surface of composite nanofibers based on high values of R^2^ and close agreement of the theoretical adsorption capacity with the experimental values.

### 3.4. Adsorption Mechanism and Reusability Test

**Figure 9:**
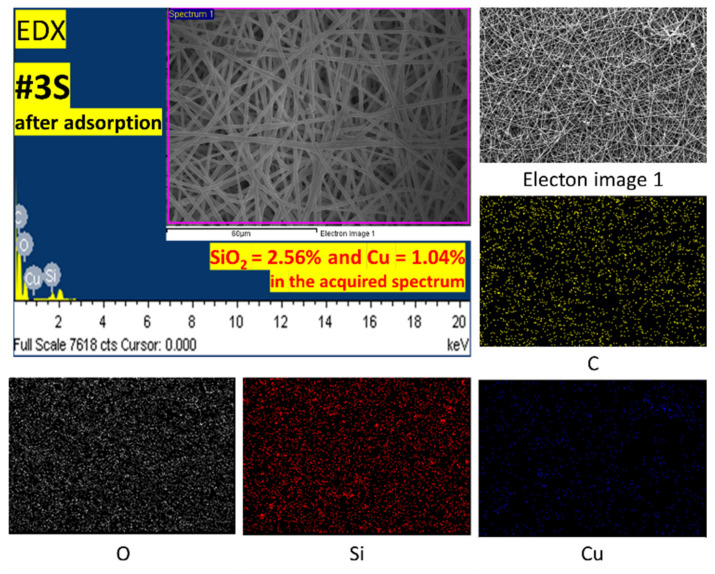
The EDX results of #3S after adsorption of Cu^2+^ ions with elemental mapping of Carbon (C), Oxygen (O), Silica (Si), and Copper (Cu).

Following the adsorption study, characterizations were carried out on the PLA/PEG-PPG-PEG/SiO_2_ nanofibrous adsorbents using FE-SEM and EDX with elemental mapping analysis, as shown in Figure 9, to verify the bonding of Cu^2+^ ions onto the nanofibrous adsorbents. The EDX result of the adsorbed #3S could be confirmed through Figure 9. After adsorption, it was confirmed that about 1.04% of Cu^2+^ ions were contained. In addition, it was confirmed that the content of SiO_2_ was 2.56%.

**Figure 10:**
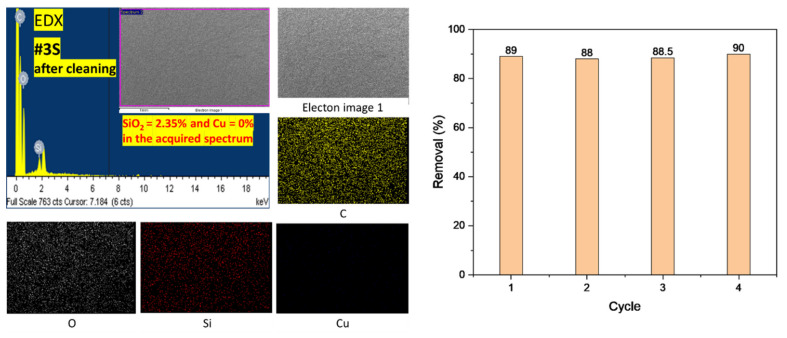
The EDX results of #3S after washing of Cu^2+^ with elemental mapping of Carbon (C), Oxygen (O), Silica (Si), and Copper (Cu) at left side, and the recyclability of the #3S at right side.

EDX was checked after washing #3S for reusability test. It was confirmed that Cu^2+^ was completely cleaned from the nanofibrous adsorbents surface, as shown in the EDX result in Figure 10; the adsorption capacities remained above ~88% after four cycles, as illustrated at right side of Figure 10. As a result of EDX analysis of the #3S sample, Si atoms were found, which means that the nanofibers were not eluted from the inside to the outside. Additionally, since Cu atoms were not found, it can increase the expectation of recycling. In addition, as a result of repeating four times, it was confirmed that the removal rate of Cu^2+^ was still expressed at 88% or more.

## 4. Conclusions

The primary objective of this research was to prepare the PLA/PEG-PPG-PEG/SiO_2_ nanofibrous adsorbents functionalized with various SiO_2_ and then to investigate their potential for Cu^2+^ ions adsorption from an aqueous medium systematically. The adsorption capacity strength of Cu^2+^ ions is affected by both the surface and bulk polymer properties of adsorbents. Therefore, the hydrophilicity of PLA was achieved by PEG-PPG-PEG blending, and the adsorption capacity was enhanced by SiO_2_ by enhancing the surface and bulk properties of polymeric adsorbents. The pH solution, contact time, and initial concentrations were all affected by the adsorption process. Based on the well-fitted PSO kinetic model, the adsorption rate was fast and exhibited high kinetic performances. The equilibrium adsorption data demonstrated that the Langmuir model was best suited to describe the adsorption of Cu^2+^ ions by nanofibers. The maximum adsorption capacities of Cu^2+^ ions on the #3S adsorbent were calculated to be ~19 mg/g. Silicon dioxide’s Si-O functional groups may be responsible for the increased adsorption rate of Cu^2+^ ions. This study demonstrated that Cu^2+^ ions could be successfully removed via adsorption by the fabricated materials. Furthermore, in terms of reusability, the current work is clearly much simpler and greener than conventional processes, with more than 88% removal capacity. As a result, the PLA/PEG-PPG-PEG/SiO_2_ nanofibrous adsorbents have a high potential for efficient adsorption of Cu^2+^ ions from wastewaters at a suitable protocol.

## Figures and Tables

**Table 1:** The blending conditions of dope solutions for preparing PLA/PEG-PPG-PEG/SiO_2_ nanofibrous adsorbents.

Sample Name of Electro-spun Nanofibrous Adsorbents	PLA Concentration (%*w*/*v*)	PEG-PPG-PEG Concentration (%*w*/*v*)	SiO_2_ Concentration (%*w*/*w*) in PLA: PEG-PPG-PEG (4:1) Solution
#0S	12	9	0
#1S			1
#2S			2
#3S			3
#4S			4
#8S			8

**Table 2:** List of kinetic and isothermal adsorption models.

Model	Equation	Plot	Ref.
Kinetic model *			
Pseudo-first-order	$\ln(Q_{e}-Q_{t})=\ln Q_{e}-\frac{k_{1}}{2.303}t$	$\ln(Q_{e}-Q_{t})\mathrm{vs.}\;t$	[31]
Pseudo-second-order	tQt=1k2 Qe2+tQe	tQtvs. t	[31]
Elovich	$Q_{t}=\frac{\ln\left(\alpha \beta \right)}{\beta }+\frac{\ln t}{\beta }$	$Q_{t\;}\mathrm{vs.}\;lnt$	[32]
Power function	$\ln Q_{t}=\ln b+k_{f}\ln t$	$\ln Q_{t}\;\mathrm{vs.}\;\ln t$	[33]
Intraparticle diffusion	$Q_{t}=c+k_{id}t^{0.5}$	$Q_{t}\;\mathrm{vs.}\;t^{0.5}$	[34]
Isotherm model **			
Langmuir	$\frac{C_{e}}{Q_{ec}}=\frac{1}{Q_{m}{\;K}_{L}}+\frac{C_{e}}{Q_{m}}$	$\frac{C_{e}}{Q_{ec}}{\;\mathrm{vs.}\;C}_{e}$	[35]
Freundlich	${\log\;Q}_{ec}=\frac{1}{n}\log C_{e}+\log K_{F}$	${\log\;Q}_{ec}\;\mathrm{vs.}\log C_{e}$	[34]
Temkin	$Q_{ec}=\mathrm{BlnA}+\;\mathrm{Bln}C_{e}$	$Q_{ec}\;\mathrm{vs.}\;\ln C_{e}$	[31]

Where; * $Q_{t}$ = time dependent adsorption capacity, $Q_{e}$ = adsorption capacity calculated at equilibrium time, $k_{1}$ = PFO constant, $k_{2}$ = PSO constant, t = time interval, α = rate of initial adsorption (mg/g. min), β = desorption constant, b = rate constant, $k_{f}$ = rate coefficient value (mg/g. min), $k_{id}$ = rate of diffusion constant (mg/g. min^0.5^), c = diffusion constant. ** $Q_{ec}$ = concentration dependent adsorption capacity, $Q_{m}$ = adsorption maximum capacity, $C_{e}$ = equilibrium concentration of ${\mathrm{Cu}}^{2+}$ in aqueous solution, $K_{L}$ = constant of Langmuir isotherm (L/mg), $K_{F}$ = constant of Freundlich isotherm (mg/g) (dm^3^/g) ^n^, n = adsorption intensity constant, A = equilibrium binding constant of Temkin isotherm (L/g), and B = equilibrium adsorption heat constant.

**Table 3:** Linear expressions of error functions.

Standard Error	Equation	Ref.
The standard error of estimate (SEE)	$SEE=\overset{n}{\underset{i=1}{\sum }}{\left(Q_{e}^{exp}-Q_{e}^{model}\right)}^{2}$	[36,37]
Coefficient of determination (R^2^)	$R^{2}=\frac{{\left(Q_{e}^{exp}-{\bar{Q}}_{e}^{model}\right)}^{2}}{\sum {\left(Q_{e}^{exp}-{\bar{Q}}_{e}^{model}\right)}^{2}+{\left(Q_{e}^{exp}-Q_{e}^{model}\right)}^{2}}$	[32]

Where $Q_{e}^{exp}$ = Experimental adsorption capacity and $Q_{e}^{model}$ = calculated adsorption capacity at equilibrium computed through model.

**Table 4:** The quantification of kinetics model parameters such as PFO, PSO, Elovich, Power function, and Intraparticle diffusion.

Kinetics Models	Parameter	#0S	#1S	#2S	#3S	#4S	#8S
Pseudo-first-order (PFO)	k_1_	0.001863442	0.0021	0.0003	0.0015	0.0011	0.002
	Q_e_	0.434461381	0.7255	0.99	0.373	0.55	0.376
	R^2^	0.154773071	0.43	0.0267	0.133	0.165	0.159
	SEE	0.386	0.216	0.1526	0.3325	0.216	0.414
Pseudo-second-order (PSO)	k_2_	0.0316	−0.0193	−1.5966	0.0874	0.039	−0.0250
	Q_e_	3.9242	3.9425	5.5906	7.0332	6.8906	5.4547
	R^2^	0.9965	0.9924	0.9979	1.0000	0.9991	0.9984
	SEE	3.1037	4.5408	1.6874	0.2005	0.901	1.4762
Elovich	α	2.6709	6.8989	6.9980	4.8554	4.7240	9.4631
	β	1.7364	1.3057	1.1462	0.8855	0.9200	1.0885
	R^2^	0.6871	0.4971	0.5494	0.8575	0.7286	0.5164
	SEE	0.9122	1.8083	1.8550	1.5908	1.5572	2.0871
Power function	K_f_	0.2041	0.2464	1.0000	0.3128	0.3065	0.2793
	B	1.3779	1.6573	1.0000	1.6296	1.6167	1.7628
	R^2^	0.6879	0.5609	1.0000	0.7084	0.7056	0.5879
	SEE	0.3227	0.5117	0	0.4711	0.4646	0.5488
Intraparticle diffusion	K_id_	0.1146	0.1021	0.1435	0.2127	3.6935	0.1339
	c	1.9605	3.5351	3.6993	3.8593	0.2087	4.2687
	R^2^	0.3569	0.1158	0.1948	0.3421	0.3522	0.1437
	SEE	1.3078	2.3978	2.4795	2.5077	2.4059	2.7771

**Table 5:** Calculated isotherm model parameters.

Isotherm Models	Parameters	#0S	#1S	#2S	#3S	#4S	#8S
Langmuir	$Q_{EL}^{meas}$	0.1217	9.9897	12.5628	19.3290	16.9314	0.0283
	K_L_	−0.0183	0.0425	0.0225	0.0219	0.0192	12.2638
	R^2^	0.6755	0.9758	0.8681	0.8849	0.8279	0.9234
	SEE	5.5	2.5786	5.0406	2.9690	4.3124	3.8200
Freundlich	K_F_	28.2586	4.5949	5.5356	5.1851	4.9133	5.8857
	n	−1.3236	9.4395	11.5455	5.5873	6.3083	12.3893
	R^2^	0.6824	0.7025	0.2469	0.6704	0.4868	0.3092
	SEE	0.3963	0.0588	0.1446	0.1182	0.1500	0.1172
Temkin	A	0.0015	3.9	6.12	2.02	2.23	8.2
	B	−1.0302	1.27	1.39	2.45	2.11	1.31
	R^2^	0.6730	0.8	0.6	0.77	0.69	0.62
	SEE	1.4667	1.75	3.25	3.9	4.1	3.0

Where $Q_{EC}^{exp}$ for #0S, #1S, #2S, #3S, #4S, and #8S were 0.9, 9, 13, 19.5, 18.1, and 12 mg/g, respectively. $Q_{EL}^{meas}$ = adsorption capacity calculated from the Langmuir model.

## Data Availability

Not applicable.
